# Diversity of coronavirus in bats from Eastern Thailand

**DOI:** 10.1186/s12985-015-0289-1

**Published:** 2015-04-11

**Authors:** Supaporn Wacharapluesadee, Prateep Duengkae, Apaporn Rodpan, Thongchai Kaewpom, Patarapol Maneeorn, Budsabong Kanchanasaka, Sangchai Yingsakmongkon, Nuntaporn Sittidetboripat, Chaiyaporn Chareesaen, Nathawat Khlangsap, Apisit Pidthong, Kumron Leadprathom, Siriporn Ghai, Jonathan H Epstein, Peter Daszak, Kevin J Olival, Patrick J Blair, Michael V Callahan, Thiravat Hemachudha

**Affiliations:** World Health Organization Collaborating Centre for Research and Training on Viral Zoonoses, King Chulalongkorn Memorial Hospital, Faculty of Medicine, Chulalongkorn University, Bangkok, Thailand; Faculty of Forestry, Kasetsart University, Bangkok, Thailand; Department of National Parks, Wildlife and Plant Conservation, Bangkok, Thailand; Inter-Department Program of Biomedical Sciences, Faculty of Graduate School, Chulalongkorn University, Bangkok, Thailand; Royal Forest Department, Bangkok, Thailand; EcoHealth Alliance, New York, USA; Naval Medical Research Center-Asia, Singapore, Singapore; Massachusetts General Hospital, Boston, MA USA

**Keywords:** Coronavirus, Bats, Diversity, Eastern, Thailand

## Abstract

**Background:**

Bats are reservoirs for a diverse range of coronaviruses (CoVs), including those closely related to human pathogens such as Severe Acute Respiratory Syndrome (SARS) CoV and Middle East Respiratory Syndrome CoV. There are approximately 139 bat species reported to date in Thailand, of which two are endemic species. Due to the zoonotic potential of CoVs, standardized surveillance efforts to characterize viral diversity in wildlife are imperative.

**Findings:**

A total of 626 bats from 19 different bat species were individually sampled from 5 provinces in Eastern Thailand between 2008 and 2013 (84 fecal and 542 rectal swabs). Samples collected (either fresh feces or rectal swabs) were placed directly into RNA stabilization reagent, transported on ice within 24 hours and preserved at −80°C until further analysis. CoV RNA was detected in 47 specimens (7.6%), from 13 different bat species, using broadly reactive consensus PCR primers targeting the *RNA-Dependent RNA Polymerase* gene designed to detect all CoVs. Thirty seven alphacoronaviruses, nine lineage D betacoronaviruses, and one lineage B betacoronavirus (SARS-CoV related) were identified. Six new bat CoV reservoirs were identified in our study, namely *Cynopterus sphinx, Taphozous melanopogon, Hipposideros lekaguli, Rhinolophus shameli, Scotophilus heathii* and *Megaderma lyra*.

**Conclusions:**

CoVs from the same genetic lineage were found in different bat species roosting in similar or different locations. These data suggest that bat CoV lineages are not strictly concordant with their hosts. Our phylogenetic data indicates high diversity and a complex ecology of CoVs in bats sampled from specific areas in eastern regions of Thailand. Further characterization of additional CoV genes may be useful to better describe the CoV divergence.

## Background

Following the Severe Acute Respiratory Syndrome (SARS) pandemic in 2002–03, caused by the SARS coronavirus (SARS-CoV), intensive surveillance has detected a great diversity of CoVs throughout the animal kingdom, especially in bats. The initial discovery of CoVs in bats was made in China following the SARS outbreak [1-5]. The emergence of the Middle Eastern Respiratory Syndrome (MERS) in 2012 renewed interest in bat-originated CoVs. The molecular investigation in Saudi Arabia revealed one *Taphozous perforatus* bat whose virus showed 100% nucleotide identity to the MERS virus found in the human index case [6]. Other subsequent studies have found MERS-related CoV lineages from a variety of bat species globally [7-11]. CoVs are divided into four genera: *Alphacoronavirus* and *Betacoronavirus* which largely infect mammals; and *Gammacoronavirus* and *Deltacoronavirus* which primarily infect avian species [12]. CoVs in bats are generally of the *Alpha*- and *Betacoronavirus* genus*,* and have been identified in bats of various species from around the world. Thailand is home to 139 different bat species, of which two are endemic species including *Hipposideros pendleburyi* and *Murina balaensis* (new species of genus *Murina*) [13,14], however CoV surveillance has only been conducted on 25 (18%) of these species [15]. The first report of bat CoVs in Thailand examined a total of 256 fecal specimens and discovered 28 positive samples in *H. larvatus* and *H. armiger* [15]. Recently in a study in Ratchaburi province, Thailand, we discovered lineage C betacoronavirus in dry bat guano fertilizer, however the bat species was not identified as specimens were collected from a mixed species roost [11]. As a result of the risk CoVs pose to human health, ecological studies of CoVs in bats are warranted, particularly to understand the baseline viral diversity circulating in wildlife hosts. Here we describe a comprehensive study of CoV diversity and prevalence among bats in Eastern Thailand to explore CoV infections in bat populations.

## Methods

Bats were captured with permission from the Department of National Parks, Wildlife and Plant Conservation. The Institutional Animal Care and Use Committee at the University of California, Davis (protocol number: 16048) approved the capture and sample collection. Bats’ species were identified in the field by experienced Thai mammalogist (PD) based on their external morphological characteristics as described by Lekagul & McNeely [16,17]. Fresh bat fecal pellets were individually stored in 0.5 ml of RNAlater® RNA Stabilization Reagent (Qiagen, Germany) while each rectal swab was placed into 1 ml of NucliSens® Lysis Buffer (bioMérieux, France), and then stored at −80°C until further analysis. Samples were examined using broadly reactive consensus hemi-nested Reverse Transcription PCR (RT-PCR) with degenerate PCR primers designed to detect all CoV lineages, targeting the *RNA-dependent RNA polymerase* (*RdRp*) gene [18]. Amplification product was visualized using 2% agarose gel electrophoresis. The *RdRP* PCR product was sequenced directly using an automated ABI PRISM 377 model sequencer or cloned using the pGEM®-T Easy Vector System before sequencing. Initially, to assess clonal sequence diversity, ten individual clones from each specimen were sequenced. Sequences were edited using Bio-edit program. Sixty-three CoV sequences obtained from 47 specimens (more than one sequence was found from 4 specimens) were deposited in GenBank with accession numbers [KJ020577 to KJ020636, KJ652018, KJ868721 and KJ868722]. Phylogenetic trees were constructed based on 353 bp *RdRp* gene sequence, corresponding to nucleotides 14,355 - 14,707 in Human CoV 229E genome (GenBank accession no. AF304460) using the maximum likelihood method.

## Results

**Figure 1:**
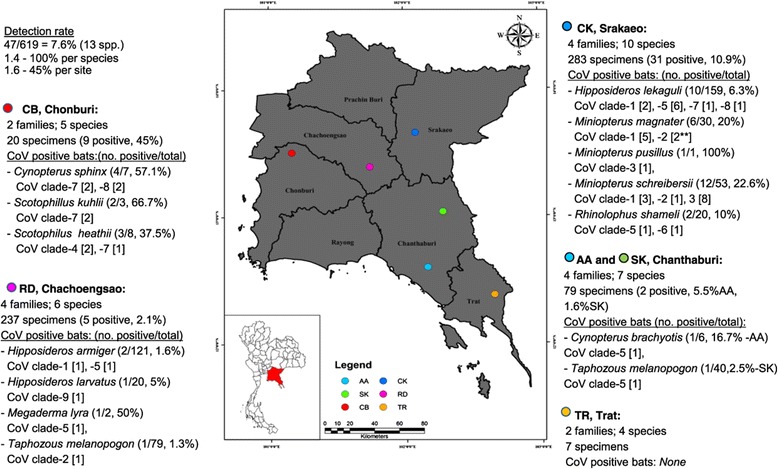
Areas in Eastern Thailand where samples were collected with CoV-positive bat species additionally named. Chakarn cave (CK, Dark blue) in Srakaeo province; Rad cave (RD, Pink) in Chachoengsao province; Chonburi province (CB, Red); Ang Aed (AA, Pale blue) and Sarika (SK, Green) caves in Chanthaburi province; and Trat province (TR, Orange). At CK site, bats were captured 4 times: in May 2008, July 2008, January 2012 and May 2012; RD site, 5 times: May 2008, July 2008, January 2012, May 2012 and January 2013; SK site, 2 times: December 2011 and May 2012; AA site, 1 time: December 2011; TR site, 1 time: December 2011. Between January and December 2012 at CB site, bats were captured monthly at 2 local swine farms. Generally, bats were caught in mist nets or harp traps as they emerged from their roosts. At two sites (CB and TR), bats were trapped during the night as they foraged near open orchards. The number of CoV positive bats [bracket] in each clade is indicated for each site. ** Sample No. BRT55593 (*Miniopterus magnater*) contained 2 different CoV species belong to clade 1 and 2.

**Figure 2:**
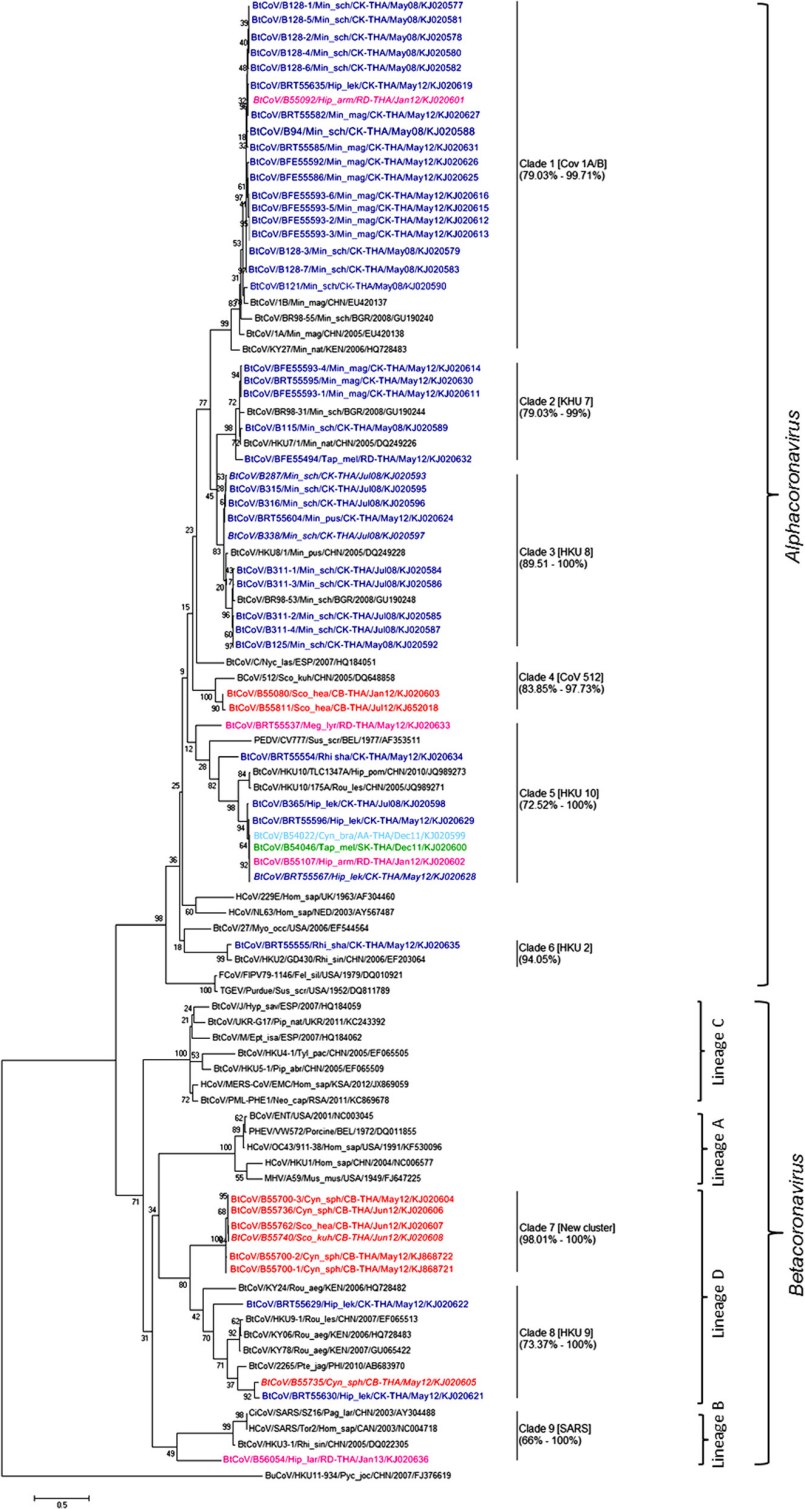
Phylogenetic trees of the coronavirus (CoV) *RNA-dependent RNA polymerase* (*RdRp*) gene at the nucleotide level. Maximum-likelihood tree of a 353 bp fragment of the *RdRp* gene from bat CoVs found in this study are colored according to their roost (Dark blue = Chakarn cave, CK; Pink = Rad cave, RD; Red = Chonburi province, CB; Pale blue = Ang Aed, AA; Green = Sarika cave, SK) and previously found in bats and other animals (black). A Bulbul deltacoronavirus HKU11-934 was used as outgroup. Alignments were constructed using Multiple Alignment Fast Fourier Transform, MAFFT. Bootstrap values were determined using 1000 replicates via MEGA 5. The tree was visualised using the FigTree program, version 1.4.0. Taxa are named according to the following pattern: identification code/strain or isolate/typical host/country/collection year/accession number. Cyn_bra, *Cynopterus brachyotis*; Cyn_sph, *Cynopterus sphinx*; Tap_mel, *Taphozous melanopogon*; Hip_arm, *Hipposideros armiger*; Hip_lar, *Hipposideros larvatus*; Hip_lek, *Hipposideros lekaguli*; Meg_lyr, *Megaderma lyra*; Rhi_sha, *Rhinolophus shameli*; Min_mag, *Miniopterus magnate*r; Min_pus, *Miniopterus pusillus*; Min_sch, *Miniopterus schreibersii*; Sco_kuh, *Scotophilus kuhlii*; Sco_hea, *Scotophilus heathii*. There were 7, 4, 6, and 3 different sequences obtained from samples no. B128 (B128-1 to B128-7), B311 (B311-1 to B311-4), BFE55593 (BFE55593-1 to BFE55593-6, and B55700 (B55700-1 to B44700-3), respectively. Representative sequences where the same exact CoV species (>99% nucleotide similarity) was found in different individuals of the same bat species at the same site show in italic. Clades 1–6 of alphacoronavirus were categorized based on the CoVs previously reported in China; bat-CoV1A/B, −HKU7, −HKU8, −CoV512, −HKU10 and -HKU2, respectively while clade 7–8 and 9 of betacoronavirus were categorized based on HKU9 and SARS CoV, respectively. The percent nucleotide similarity within each clade is shown in parentheses under the clade name.

A total of 626 bats representing 19 species (Table 1) were sampled (84 fecal and 542 rectal swab specimens) between 2008 and 2013 from 6 locations in 5 of 7 provinces in Eastern Thailand. CoV RNA was detected in 47 (7.6%) specimens (17 fecal samples and 30 rectal swabs) from 13 different bat species. Detection rates for bat CoVs were 1.6% to 45% per site in 5 of the sampling sites (Figure 1). Phylogenetic analysis of nucleotide sequences of 353 bp *RdRp* gene showed that 37 samples were members of the *Alphacoronavirus* genus and 10 belonged to the *Betacoronavirus* genus. The phylogenetic reconstruction showed 9 different clades of bat CoVs (Figure 2). There were 6 clades in alphacoronavirus (clades 1–6), 2 in lineage D betacoronavirus (clades 7 and 8) and 1 in lineage B betacoronavirus (clade 9). The six alphacoronavirus clades were divergent, but were related to CoVs previously identified in bats from China, Bulgaria and Kenya [19-21]. The percent nucleotide similarity within each clade was calculated and is included in Figure 2.

**Table 1:** **Bat species tested for coronaviruses**

**Family**	**Species**	**No. of positive/total‡ (%)**	**Sampling site (year)†**	**CoV clade(s) [cluster]/(no. positive)**
**Pteropodidae**	*Cynopterus brachyotis*	1/9 (11.1)	AA(2011*); TR(2011)	5 [HKU10]/(1)
	***Cynopterus sphinx***	4/14 (28.6)	AA(2011); RD(2008); TR(2011); CB(2012*)	7 [New cluster]/(2), 8 [HKU9]/(2)
	*Eonycteris spelaea*	0/11 (0)	AA(2011); TR(2011); CB(2012)	
	*Rousettus amplexicaudatus*	0/3 (0)	SK(2011)	
**Emballonuridae**	*Taphozous longimanus*	0/12 (0)	RD(2008/2012)	
	***Taphozous melanopogon***	2/123(1.6)	RD(2012*/2013); CK(2012); SK(2011*/2012)	2 [HKU7]/(1), 5 [HKU10]/(1)
**Hipposideridae**	*Hipposideros armiger*	2/140(1.4)	CK(2012); RD(2008/2012*/2013); SK(2012)	1 [CoV1A/B]/(1), 5 [HKU10]/(1)
	*Hipposideros cineraceus*	0/3	CK(2012)	
	*Hipposideros larvatus*	1/29(3.4)	CK(2012); RD(2008/2012/2013*)	9 [SARS]/(1)
	***Hipposideros lekaguli***	10/159(6.3)	CK(2008*/2012*)	1 [CoV1A/B]/(2), 5 [HKU10]/(6), 8 [HKU9]/(2)
**Macroglossinae**	*Macroglossus sobrinus*	0/2(0)	AA(2011); TR(2011)	
**Megadermatidae**	***Megaderma lyra***	1/2 (50)	RD(2012*/2013)	5 [HKU10]/(1)
**Rhinolophidae**	***Rhinolophus shameli***	2/20(10)	CK (2012*)	5 [HKU10]/(1), 6 [HKU2]/(1)
**Vespertilionidae**	*Miniopterus magnater*	6/30(20)	CK (2012*)	1 [CoV1A/B]/(5), 2 [HKU7]/(2**)
	*Miniopterus pusillus*	1/1(100)	CK(2012*)	3 [HKU8]/(1)
	*Miniopterus schreibersii*	12/53(22.6)	CK(2008*/2012)	1 [CoV1A/B]/(3), 2 [HKU7]/(1), 3 [HKU8]/(8)
	*Myotis horsfieldii*	0/4(0)	CK(2012); CB(2012)	
	*Scotophilus kuhlii*	2/3(66.7)	CB(2012*)	7 [New cluster]/(2)
	***Scotophilus heathii***	3/8(37.5)	CB(2012*)	4 [CoV 512]/(2), 7 [New cluster]/(1)
	Total	47/626(7.5)		

**‡**Samples were 84 fecal and 542 rectal swabs; *A positive location (and year) is indicated by an asterisk; First report of CoV in species (indicated in bold).

**†**AA = Ang Aed, Chataburi; CB = Chonburi; CK = Chakan, Srakaew; RD = Rad, Chachongsao; SK = Sarika, Chantaburi; TR = Trat.

**Sample No. BRT55593 (*Miniopterus magnater*) contained 2 different CoV species belong to clade 1 and 2.

In our study, host restriction of CoVs was demonstrated in clade 4 (CoV512) and clade 6 (HKU2). Clade 4 CoV from *Scotophilus heathii* was clustered with bat CoV512 previously found in *S. kuhlii* from China [4]. Clade 6 CoV (BRT55555) found in *Rhinolophus shameli,* was clustered with HKU2 (*R. sinicus*) described in China [22]. This is in accordance with previous studies which have demonstrated that individual CoVs are associated with a single species or genus including *Carollia, Eptesicus, Miniopterus*, and *Rhinolophus* bats [4,19,20,23].

We found evidence for species of CoV in almost every clade in this study being shared by different bat species from different families. For example clades 1–3 CoVs were found in 3 bat families, in the *Miniopterus magnater*, *M. schreibersii*, *M. pusillus, H. lekaguli*, *H. armiger* and *T. melanopogon*. Similarly, clade 5 CoVs were found in 6 bat species (5 genera, 5 families) from 4 different sites, clade 7 CoVs were found in 3 bat species captured from the same location and clade 8 CoVs were found in *Cynopterus sphinx* (fruit bat) and the insectivorous *H. lekaguli* (Figure 2). Further, many CoVs species were found in a single bat species such as the *H. lekaguli, R. shameli, T. melanopogon*, and *S. heathii* (Figure 2). For example, 10 *H. lekaguli* bats roosting in the same colony were found to harbor 2 lineage D betacoronaviruses and 8 alphacoronaviruses.

Seven CoVs in clade 7 from *S. kuhlii, S. heathii* and *C. sphinx* were clustered in an independent lineage. These viruses (from 5 bats) had 99.15-100% identity of 119 amino acids and differed from HKU9 by 16.11-16.95% (Figure 2). Further analyses using longer gene fragments and other genes from greater number of bats are required for confirmation of this novel group.

One specimen from *M. magnater* (BFE55593) was found to be co-infected with 2 CoV species. Further, three individual bats from 2 species (samples no. B128 [*M. schreibersii*], B311 [*M. schreibersii*], and B55700 [*C. sphinx*]) were found to be co-infected with multiple strains of the same CoV species. Initial sequencing showed multiple nucleotide peaks upon direct sequencing chromatogram. These PCR products were cloned and 10 individual clones were sequenced in order to assess clonal sequence diversity. Analysis of 353 bp sequences revealed the presence of sequence variants within single samples (also known as quasispecies [24]). There were 7, 4, 6, and 3 different sequences obtained from samples no. B128, B311, BFE55593, and B55700, respectively. All sequences from individual samples were clustered into the same CoV species (i.e. represented CoV quasispecies) except in BFE55593, where 2 sequences were clustered to clade 2 and the other 4 sequences were clustered into clade 1.

In addition, this is the first report describing the presence of CoV RNA in 6 bat species including *C. sphinx*, *T. melanopogon*, *H. lekaguli*, *R. shameli*, *S. heathii* and *Megaderma lyra,* where the latter is the newly reported bat family (Megadermatidae) found to harbor CoV.

## Discussion and conclusion

Data from this study demonstrates that CoV infection in bats sampled in Eastern Thailand is not uncommon and infection is distributed among a range of species. The CoVs found in bats from this small region of Thailand were genetically related to bat CoVs found in several countries from different regions of the world such as China, Philippines, Kenya, Spain and Bulgaria [18-21,25]. MERS-like CoV (previously found in environmentally sampled bat feces from Ratchaburi province, Western Thailand [11]) was not found in this study, despite sampling the bat genus *Taphozous*, a likely MERS-CoV reservoir found in Saudi Arabia [6], in our study. Further studies on individual bat species in Western Thailand to identify bat reservoirs for MERS- or SARS-like CoVs may be justified. Phylogenetic analysis revealed close correlations between CoV/B56054 from *H. larvatus* and SARS-like CoV belonging to lineage B betacoronavirus from *Rhinolophus* in China [1]. This finding was in accordance with the previous bat CoV study in Thailand [15]. However three bat species (*H. lekaguli*, *M. lyra*, and *M. schreibersii*) and 3 bat families (Pteropodidae, Emballonuridae, and Rhinolophidae) previously reported negative for CoV in Thailand [15], were positive for CoVs in our study (Table 1).

At several of our sites, we observed many different bat species from different families roosting in the same cave and subsequently were found to be harboring the same bat CoV species. For example, *H. lekaguli* harbored CoVs of the same genetic lineage as *Miniopterus* CoV in clades 1, and *R. shameli* in clade 5. Co-roosting of theses bats in an enclosed cave environment may have facilitated the exchange of viruses. Three bat species (*S. kuhlii*, *S. heathii* and *C. sphinx*) captured from the same location (unknown roost) carried similar viruses clustered in clade 7. The spatial overlap at feeding areas and temporary night roosts for *S. kuhlii*, *S. heathii* (insectivorous) and *C. sphinx* (nectarivorous) may have facilitated the exchange of viruses, as they may not necessarily be co-roosting diurnally and do not share the same direct food source. This data supports previous studies in Spain, China and South America, where different bat species sampled within the same location carried similar viruses [4,25,26]. However, more research is needed to understand interspecific bat behavior and transmission potential for these species with seemingly different diet and foraging patterns.

Further, the *H. armiger* and *T. melanopogon*, which roosted at a different colony from the *Miniopterus* bats, also harbored CoV of the same genetic lineage as the *Miniopterus* CoV in clades 1 and 2 respectively. Similarly, HKU10-related CoVs (clade 5) were found in 5 divergent bat families including Megadermatidae, Pteropodidae, Emballonuridae, Hipposideridae, and Rhinolophidae (Figure 2). These HKU10- positive bat species, *H. lekaguli* (CK site), *H. armiger* (RD), *C. brachyotis* (AA), *T. melanopogon* (SK), *M. lyra* (RD), and *R. shameli* (CK), were from 4 different sampling sites. Interestingly, these viruses were closely related to HKU10 CoVs found in *Rousettus leschenaulti* and *H. pomona* in China, where interspecies transmission between bats of different suborders was also demonstrated [27]. Further studies and deeper characterization of these bat CoVs infecting different bat species may provide additional insight to their host range and the evolutionary history of their interspecies transmission. These findings indicate a greater diversity and higher ecological complexity of bat CoVs in Eastern Thailand than previously appreciated.

Two or more different CoV clades/lineages were also found circulating in the same bat species from the same site, for example *C. sphinx*, *H. armiger*, *H. lekaguli*, *R. shameli*, *M. schreibersii*, *M. magnater,* and *S. heathii*, and from different roosts for *T. melanopogon* (Table 1 and Figure 2). This CoV diversity may be associated with bat migration or bats from different species sharing foraging sites. Previous studies also found evidence of cross-species transmission in bats, for example *Artibeus lituratus* from Mexico [23], and *R. sinicus* [28] and *R. leschenaulti* from China [2].

Interestingly, co-infection of divergent CoV lineages was found in one bat (*M. magnater*, BFE55593), which was infected with 2 different CoV species, clustered in clades 1 (CoV 1A/B) and 2 (HKU7) (Figure 2). This finding is similar to a previous report from China, where co-infection of bat CoV 1B and HKU8 were detected in *M. pusillus* using species-specific RT-PCR assays [29]. Co-infection with different CoVs in the same host may facilitate recombination between these CoVs. Further studies of co-infection and CoV recombination within a given bat host could improve our understanding on the evolution of CoVs, including specific mutations or recombination events (e.g. involving the Spike gene), that could facilitate spillover to novel species.

In conclusion, phylogenetic analysis of our study revealed a high genetic diversity of CoVs and presence of cross species dissemination in bats from the Eastern region of Thailand. Finding of new viral reservoirs and the putative novel betacoronavirus lineage in this study emphasizes the need for additional CoV surveillance. Our data can serve as an additional dataset to the global surveillance of emerging CoVs, which may include potentially harmful pathogens to human health. In order to have a complete understanding of the ecology and transmission of CoV, a comprehensive analysis of bats across their migratory routes in Africa, Southeast Asia and Australia should be conducted.

## Abbreviations

CoV: Coronavirus
MERS: Middle East Respiratory Syndrome
SARS: Severe Acute Respiratory Syndrome
RT-PCR: Reverse transcription-polymerase chain reaction
